# Missed opportunity for alcohol use disorder screening and management in primary health care facilities in northern rural Tanzania: a cross-sectional survey

**DOI:** 10.1186/s13011-022-00479-x

**Published:** 2022-07-06

**Authors:** Dorothy Mushi, Candida Moshiro, Charlotte Hanlon, Joel M. Francis, Solomon Teferra

**Affiliations:** 1 Department of Psychiatry and Mental Health, Muhimbili University of Health and Allied Sciences, Dar es Salaam, Tanzania; 2 Department of Psychiatry, School of Medicine, College of Health Sciences, Addis Ababa University, Addis Ababa, Ethiopia; 3 Centre for Innovative Drug Development and Therapeutics Trial for Africa (CDT-Africa) College of Health Science, Addis Ababa University, Addis Ababa, Ethiopia; 4 Department of Epidemiology and Biostatistics Muhimbili, University of Health and Allied Sciences, Dar es Salaam, Tanzania; 5 Centre for Global Mental Health, Health Service and Population Research Department, Institute of Psychiatry, Psychology, and Neuroscience, King’s College London, London, UK; 6 Department of Family Medicine and Primary Care, Witwatersrand University, Faculty of Health Sciences, Johannesburg, South Africa

**Keywords:** Alcohol use disorder, Primary health care, Screening, Detection, Alcohol use disorder management, Help-seeking, Barriers to seeking care

## Abstract

**Objective:**

The study aimed to identify the missed opportunity for detection and management of alcohol use disorder by primary health care workers.

**Design:**

A cross-sectional survey

**Setting:**

Outpatient services in the six governmental primary health care facilities in Moshi district council in Tanzania.

**Participants:**

A total of 1604 adults were screened for alcohol use disorder (AUD) using the Alcohol Use Disorder Identification Test (AUDIT). Participants scoring 8 or above then provided details about their help-seeking behavior and barriers to seeking care. Participants’ records were reviewed to assess the screening and management of AUD.

**Results:**

In the last 12 months, 60.7% reported alcohol use, and heavy episodic drinking (HED) was reported by 37.3%. AUD (AUDIT ≥ 8) was present in 23.9%. Males were more likely to have HED (aPR = 1.43;95% CI:1.3 to 1.4) or AUD (aPR = 2.9; 95% CI 1.9 to 4.2). Both HED and AUD increased with age. Only one participant (0.3%) had documented AUD screening and management. Only 5% of participants screening positive for AUD had sought help. Reasons for not seeking care were thinking that the problem would get better by itself (55.0%), wanting to handle the problem alone (42.0%), or not being bothered by the problem (40.0%).

**Conclusion:**

While reported alcohol use, HED, and AUD are common among patients presenting to primary healthcare facilities in northern Tanzania, help-seeking behavior and detection are very low. Not screening for AUD in primary health care is a missed opportunity for early detection and management. There is an urgent need to develop interventions to increase the detection of AUD by health care providers, while also addressing help-seeking behavior and barriers to seeking care.

## Introduction

Alcohol use is socially accepted in most cultures worldwide. Excessive alcohol use accounts for substantial adverse consequences for health, social wellbeing, and economic status [1, 2]. Approximately one in 20 of all disability-adjusted life-years (5.1% DALYs) and 5.3% of all deaths globally are attributed to the harmful use of alcohol [1]. The mortality resulting from alcohol use is higher than that caused by other non-communicable or communicable diseases such as tuberculosis, HIV/AIDS, and diabetes [1]. Alcohol use disorder is responsible for around half of all alcohol-related harm [3].

Alcohol Use Disorder is defined as a cluster of cognitive, behavioral, and physiological symptoms indicating that the individual continues using alcohol despite significant alcohol-related problems [4]. In 2016, an estimated 283 million people aged 15 years and above had an alcohol use disorder (AUD) (5.1% of all adults). The 12-month prevalence of AUD was 3.7% in Africa in 2016 [1]. In Tanzania, the estimated prevalence of AUD varies across populations, ranging from 5.7% to 28% [5–7]. Heavy episodic drinking is also of public health concern. HED is a pattern of drinking whereby a person consumed at least 60 g or more of pure alcohol on at least one occasion in the past 30 days. Consumption of 60 g of pure alcohol corresponds approximately to six standard alcoholic drinks [1]. In 2016, the worldwide prevalence of HED was 18.2% and was associated with the different types of non-communicable as well as infectious diseases [1].

Studies conducted in healthcare facilities in sub-Saharan Africa (SSA) have shown that approximately one in five people attending healthcare facilities met the criteria for AUD [8–12]. This suggests the possibility that persons with AUD may frequently access health care services [13], though not specifically for their AUD. There is also evidence that most persons with AUD also have co-occurring acute or chronic physical or mental health problems [14–17]. Even with such a substantial magnitude of AUD and the clear adverse consequences, AUD appears to be rarely detected by health care providers [18, 19], leading to a wide treatment gap [18, 20, 21]. This gap is particularly marked in low-income and lower-middle-income countries [17, 18, 21].

Integration of evidence-based interventions for AUD into existing health care systems is recommended to address the AUD treatment gap [22–24]. Studies demonstrate that such integrated services can minimize cost and maximize the effectiveness of managing AUD and co-occurring health problems [22, 25–28]. An emerging and robust evidence base indicates the feasibility of delivering AUD interventions in primary health care settings [18, 29, 30]. However, studies have identified bottlenecks to the integration process and delivery of AUD interventions in health care systems [17, 31, 32]. These bottlenecks include low help-seeking behavior for people with AUD [17, 24, 33, 34] for example, due to stigma attached to AUD and lack of awareness. Further barriers to care exist for those who seek help [17, 24], categorized into individual, structural, and contextual factors [31, 32].

To improve detection, help-seeking, and barriers to care for AUD and consequently narrow the treatment gap for AUD, more evidence from diverse contexts is needed. The purpose of this study was to identify the missed opportunity for detection and management of AUD by primary health care workers in Tanzania. The findings will inform the adaptation and piloting of a model for the integration of AUD interventions at the primary health care delivery level [35].

## Methods

### Study design and settings

A facility-based, cross-sectional survey was conducted from September to November 2019 in the six government health centers in Moshi district council, Kilimanjaro region, Tanzania. Kilimanjaro region is located around 530 km from the main economic city of Dar es Salaam (the former capital city of Tanzania). According to Tanzania's census [36], the Moshi district council had a population of 539,586 (262,897 males and 276,689 females) in 2012. Two faith-based hospitals have been designated by the Tanzanian Ministry of Health as the district hospitals, and there are also eight health centers and 88 dispensaries. Primary health care (PHC) services in Tanzania are provided at the district level through the health centers and dispensaries. Dispensaries are the first contact point for basic health services in the community.

The study area was selected based on the existence of previous studies of alcohol use disorder in Tanzania. In these previous studies, alcohol use (68.0% to 70.0%) and problematic alcohol use (20.0% to 47.0%) were found to be highly prevalent in the community [6, 7]. Based on previous findings, it was recommended that access to interventions for AUD should be expanded [6, 7, 37, 38]. Therefore, this study was conducted to inform the adaptation of a model for integrating services for AUD in PHC in the study area [35].

### Participants and sample size

Adult outpatients (aged 18 years and above) attending PHC during the study period and willing to give consent were invited into the study. The sample size was determined based on the assumption that the prevalence of AUD would range from 10.0% to 20.0% in the PHC setting [6, 7]. We assumed a detection rate of AUD of 10.0% from a previous study conducted in five low and middle-income countries [19]. The possibility of clustering was considered at the health facility level, the following were the reasons, (i) health care providers working in one PHC are more likely to share similarities such as clinical knowledge, practices, health care delivery infrastructure, and system. (ii) Likewise, for the people who attend the same PHC, because they are living in the same area, they are more likely to share similarities in terms of their health beliefs, practices, and socioeconomic status. We could not find the published studies from Tanzania to guide the estimation of the intra-cluster correlation (ICC) for the detection of AUD in health centers. Therefore, we assumed the ICC to be 0.01, from a review of 31 studies [39], with a design effect of 1.95, which resulted in a sample size of 1600. The sample size was distributed over six PHC facilities based on the size of the catchment population. To achieve a total sample in each PHC facility, all men were recruited and a systematic sample of one in three women was recruited. Men were oversampled to ensure an adequate sample size that aimed for equal representation of men and women, as more women than men attend PHC, with an approximate ratio of men to women of 1:3.

### Data collection and variables

The collection of data through the face-to-face interview was conducted electronically, through a mobile phone or tablet using Open Data Kit (ODK) software. Data collection tools were piloted, pre-tested, and modified accordingly. This helped the research team plan for the number of interviews to be conducted by one data collector per day while minimizing interference to the provision of clinical services and extra time spent by the participant at the health facility. Interviews were conducted after the participant’s clinic appointment. This facilitated participant.

engagement and cooperation because the participant had received the treatment that brought them to the health facility. Furthermore, healthcare professionals were not biased by the research interview findings. The questionnaire was administered in Swahili by ten final-year medical students supervised by one study coordinator and the principal investigator.

### Measures

Socio-demographic variables collected in the study included age, sex, marital status, level of education, and occupation.

### Alcohol use and alcohol use disorder

The World Health Organization’s Alcohol Use Disorder Identification Test (AUDIT) was used to assess alcohol consumption during the past 12 months [40]. The AUDIT assessed alcohol consumption in terms of quantity, drinking pattern, and problems associated with alcohol use. Each item on the AUDIT was rated on a five-point Likert scale from 0 to 4, giving a total score ranging from zero to 40. The first question, which asks how often does someone have a drink containing alcohol, informed alcohol use during the past 12 months. Heavy episodic drinking was defined based on the third question which asks ‘how often do you have six or more drinks on one occasion?’ A total score of eight or more indicates a tendency to problematic drinking as follows: a score of 8–15 indicates hazardous alcohol use, a score of 16–19 indicates harmful alcohol use, and an AUDIT score of 20 and above requires further evaluation for suspected alcohol dependence [41]. In this study, for participants who had an AUDIT score of ≥ 8, data on help-seeking, and barriers to seeking care for AUD were collected. In addition, a clinical consultation checklist form that contained patient particulars, a description of the patient’s symptoms/ complaint, diagnosis, and treatment plan was completed to assess whether the PHC provider had documented the AUD diagnosis and if the patient received any AUD intervention. The estimated mean consultation time in PHC facilities is 10 min [42]

The AUDIT has been validated across different populations in low and middle-income countries’ settings [40, 43, 44]. The AUDIT has been adapted into the national language (Swahili) and used previously in Tanzania [5–7, 45]**.** The Swahili version of the AUDIT has been validated and the psychometric properties found to be acceptable [45]. For traditional brews, we applied the ethanol concentration data from the previous assessment of the locally available brews including the traditional liquor(spirit) and beers [46]. A pictorial chart demonstrating the standard drinks of the most commonly consumed traditional and industrial alcoholic beverages has been adapted [7] and was used in this study.

### Barriers to seeking care

Barriers to accessing care based on various factors, including cost, service satisfaction, logistics, and stigma, were assessed using The Barriers to Accessing CarE (BACE) questionnaire [47]. The BACE measures several barriers that may prevent individuals from seeking or continuing with care for a mental health problem. The barriers include individual and structural factors, such as individual knowledge and attitude about mental health care, social support, stigma, infrastructure, previous experience with mental health care, and treatment cost. The BACE has been adapted previously for use in low-income African countries [17, 48, 49].

### Help-seeking behavior

Awareness of having an AUD and the individual’s perspective that AUD is a problem that needs care was assessed using an adapted version of the PRIME help-seeking behavior questionnaire [50]. The questionnaire was administered to participants with an AUDIT score of ≥ 8. First, participants were asked if they had ever sought help for their alcohol use (Yes or No). Those who had sought help were then asked about the time, type, and perceived usefulness of the help they received.

### Screening and management of AUD at PHC

Any documentation of detection and/or other interventions for problematic alcohol use (not just that the person drinks alcohol) in the clinical consultation form was considered as evidence for the screening and management of AUD at PHC.

### Data management and analysis

Sociodemographic characteristics were summarized using proportions for categorical variables or median, and interquartile ranges for continuous variables. Screening and management of AUD were calculated as the proportion of participants with a probable AUD for whom there was documentation indicating detection and/or other interventions for problematic alcohol use. Odds ratios (OR) from a cross-sectional study might overestimate the effect when the outcome is common [51, 52], so prevalence ratios were obtained using a Poisson working model [53, 54]. A multivariable model was used to examine factors associated with alcohol use, HED, and AUD. All exposures (sex, age, marital status, employment, and education level) are known confounders [55, 56] and therefore were included in the multivariable models [57]. Crude and adjusted prevalence ratios with corresponding 95% confidence intervals and p-values were reported. We used weights to account for unequal selection probabilities, and adjusted for clustering and stratification (STATA *svy* commands) while calculating the prevalence of HED and AUD, and fitting regression models. [58]. A two-sided *P* < 0.05 was considered statistically significant. Data were analyzed using STATA version 13 [59].

### Ethics approval and consent to participant

This study was approved by the Addis Ababa University College of Health Science Institutional Review Board (protocol number: 023/19/psyc) and the Muhimbili University of Health and Allied Sciences Institutional Review Board (Ref.No.DA.282/298/01.C/). All participants gave written informed consent; if non-literate, participants gave a thumbprint to signify consent in presence of a data collector and another witness.

## Results

### Sociodemographic characteristics

We recruited 1615 participants, 11 refused to participate in the study, and lack of time due to other responsibilities was the reason reported by almost all of the participants. Of 1604 individuals, the median age of study participants was 41 years (interquartile range 29—56). Over half, 58.2% (*n* = 933) of participants were female. About two-thirds of participants had completed primary education, 65.0% (*n* = 607) of females and 63.0% (*n* = 425) of males. 63.7% (*n* = 595) of females and 70.5% (*n* = 473) of males were currently married. Details of sociodemographic characteristics are presented in Table 1.

**Table 1:** Socio-demographic characteristics of PHC attendees by sex, in Moshi district council, Tanzania (2019)

Characteristic	Categories	Total N (%) *n* = 1604	Sex	
			Female N (%) *n* = 933	Male N (%) *n* = 671
Age in years	< 25	225 (14.0)	157 (16.8)	68 (10.1)
	25–39	526 (32.8)	333 (35.7)	179 (28.8)
	40–49	292 (18.2)	163 (17.5)	129 (19.2)
	50–59	229 (14.3)	120 (12.9)	109 (16.2)
	≥ 60	332 (20.7)	160 (17.1)	172 (25.6)
Education level	Never went to School	84 (5.2)	44 (4.7)	40 (5.9)
	Incomplete primary school	100(6.2)	56 (6.0)	44 (6.6)
	Primary education	1032 (64.3)	607 (65.0)	425 (63.3)
	Secondary education	283 (17.6)	174 (18.7)	109 (16.2)
	University/ college	105 (6.6)	52 (5.6)	53 (7.9)
Marital status	Married	1068(66.5)	595 (63.7)	473 (70.5)
	Separated/divorced	78(4.8)	51 (5.5)	27 (4.0)
	Widowed	130(8.1)	85 (9.1)	45 (6.7)
	Cohabiting	54(3.4)	38 (4.1)	16 (2.4)
	Single	274(17.1)	164 (17.6)	110 (16.4)
Employment	Employed	183 (11.4)	84 (9.0)	99 (14.8)
	Self-employment	933 (58.2)	544 (58.8)	384 (57.2)
	Unemployed/	362 (22.6)	238 (25.5)	124 (18.5)
	Volunteered/ Student	59 (3.7)	39 (4.2)	20 (3.0)
	Retired	67 (4.2)	23 (2.5)	44 (6.6)

### Alcohol use and prevalence of AUD

Overall, 60.7% of the participants reported to use alcohol in the last 12 months (*n* = 974) (95% CI: 59.1 to 63. 8%). Using the last 12 months timeframe, the weighted prevalence of HED was 37.3% (*n* = 599), and AUD (AUDIT ≥ 8) was 23.9% (*n* = 378), (95% CI: 18.6 to 30.2%). The prevalence of AUD was 3 times higher among males 38.7% (*n* = 260) than females 13.1% (n = 118), *p*-value < 0.001. Based on the AUDIT score among those who reported alcohol use in the last 12 months, 18.0% (*n* = 281) were classified as hazardous alcohol use (AUDIT score 8–15), 3.0% (*n* = 42) harmful alcohol use (AUDIT score 16–19) and 3.0% (*n* = 55) probable alcohol dependence (AUDIT score ≥ 20).

### Factors associated with alcohol use and AUD

In the final multivariable analysis, reported alcohol use was independently associated with the male gender (aPR 1.42,95% CI:1.3 to 1.5) and older age (Table 2). Moreover, males were more likely to have HED (aPR = 1.43;95% CI:1.3 to 1.4) (Table 3) or AUD (aPR = 2.9; 95% CI 1.9 to 4.2) (Table 4). Both HED and AUD increased with age, participants aged 25 years and above had more prevalence of HED (Table 3) and AUD (Table 4) when compared to participants aged less than 25 years.

**Table 2:** Factors associated with alcohol use among PHC attendees in Moshi district council, Tanzania (2019)

Factor	Categories	Total	Reported AU n (%)	Crude PR (95% CI)	Adjusted PR (95% CI)
Age in years	< 25	86	33 (38.2)	1	1
	25–39	302	173 (57.4)	1.52(1.3–1.8) ^**^	1.43(1.2–1.7) ^**^
	40–49	196	131(67.1)	1.76(1.5–2.1) ^**^	1.58(1.3–1.9) ^**^
	50–59	165	119 (72.0)	1.91(1.6–2.3) ^**^	1.70(1.4–2.1) ^**^
	≥ 60	225	152 (67.7)	1.78(1.5–2.1) ^**^	1.54(1.3–1.9) ^**^
Education level	Never went to School	49	28(58.3)	0.91(0.7–1.1)	0.87(0.7–1.0)
	Incomplete primary	66	43(66.0)	1.03(0.9–1.2)	0.99(0.9–1.2)
	Primary	648	406(62.7)	0.96(0.8–1.1)	
	Secondary	148	77(52.3)	0.83(0.7–0.9) ^*^	0.89(0.8–1.0)
	University/ college	63	38 (60.0)	0.96(0.8–1.1)	0.96(0.8–1.1)
Sex	Female	507	383(75.5)	1	1
	Male	467	233 (50.0)	1.48(1.4–1.6) ^**^	1.42(1.3–1.5) ^**^
Marital status	Married	681	434 (63.7)	1	1
	Separated/divorced	48	30 (61.5)	0.96(0.8–1.2)	1.00(0.8–1.2)
	Widowed	86	57 (66.1)	1.02(0.9–1.2)	1.01(0.9–1.2)
	Cohabiting	28	14(51.8)	0.81(0.6–1.1)	0.98(0.8–1.2)
	Single	131	63 (47.8)	0.76(0.7–0.9) ^**^	0.90(0.8–1.0)
Employment	Unemployed	296	189 (60.7)		1
	Self-employed	131	94 (71.5)	1.15(1.0–1.3) ^*^	1.12(1.0–1.3)
	Employed	547	320 (58.6)	0.95(0.9–1.0)	0.89(0.8–1.0) ^*^

*AU* = Alcohol use, *PR* prevalence ratio,* (%) *Row percentage

^*^p < 0.05

^**^p < 0.001

**Table 3:** Factors associated with heavy episodic drinking among PHC attendees in Moshi district council, Tanzania (2019)

Factor	Categories	Total	Reported HED n (%)	Crude PR (95% CI)	Adjusted PR (95% CI)
Age in years	< 25	47	25(54.6)	1	1
	25–39	208	143(68.8)	1.29(1.0–1.6) ^*^	1.42(1.2–1.7) ^**^
	40–49	124	78 (63.2)	1.17(0.9–1.5)	1.58(1.3–1.9) ^**^
	50–59	102	63 (61.8)	1.16(0.9–1.5)	1.70(1.4–2.1) ^**^
	≥ 60	118	62 (52.4)	0.97(0.8–1.2)	1.54(1.3–1.9) ^**^
Education level	Never went to School	30	18 (61.2)	1.03(0.8–1.3)	1.07(0.9–1.3)
	Incomplete primary	37	21 (56.0)	0.92(0.7–1.2)	0.99(0.8–1.2)
	Primary	391	236 (60.3)	1	1
	Secondary	96	62 (64.8)	1.09(1.0–1.2)	1.08(0.9–1.2)
	University/ college	45	32 (71.4)	1.17(1.0–1.4)	1.14(0.9–1.4)
Sex	Female			1	1
	Male	361	257(71.2)	1.38(1.2–1.5) ^**^	1.43(1.3–1.5) ^**^
Marital status	Married	424	264(62.2)	1	1
	Separated/divorced	33	23(68.7)	1.11(0.9–1.4)	1.00(0.8–1.2)
	Widowed	36	15 (41.8)	0.68(0.5–0.9)	1.01(0.9–1.2)
	Cohabiting	20	14 (71.4)	1.14(0.9–1.5)	0.98(0.8–1.2)
	Single	86	56 (65.6)	1.06(0.9–1.2)	0.90(0.8–1.0)
Employment	Unemployed	143	69(48.3)		1
	Self-employed	89	60(67.9)	1.39(1.2–1.7) ^**^	1.12(1.0–1.3)
	Employed	367	246 (67.0)	1.38(1.2–1.6) ^**^	0.89(0.8–1.0) ^*^

*PR* prevalence ratio, *(%) *Row percentage, and *HED* heavy episodic drinking

^*^p < 0.05

^**^p < 0.001

**Table 4:** Factors associated with alcohol use disorder among PHC attendees in Moshi district council, Tanzania (2019)

Factor	Categories	Total	Reported AUD n (%)	Crude PR (95% CI)	Adjusted PR (95% CI)
Age in years	< 25	225	26 (11.6)	1	1
	25–39	526	135 (25.7)	2.4 (1.3–4.6) ^*^	(1.1–4.0) ^*^
	40–49	292	80 (27.4)	2.5 (1.4–4.4) ^*^	(1.1–3.9) ^*^
	50–59	229	79 (34.5)	3.2 (1.7–5.8) ^**^	2.6 (1.4–4.9) ^*^
	≥ 60	332	58 (17.5)	1.6 (0.8–3.2)	1.4 (0.6–3.1)
Education level	Never went to School	84	19 (22.6)	0.9 (0.5–1.7)	0.9 (0.6–1.3)
	Incomplete primary	100	18 (18.0)	0.7 (0.5–0.9) ^*^	0.8 (0.6–1.1)
	Primary	1032	254 (4.6)	1	1
	Secondary	283	63 (22.3)	0.9 (0.7–1.3)	0.9 (0.7–1.4)
	University/ college	105	24 (22.9)	(0.6–1.5)	0.8 (0.6–1.2)
Sex	Female	933	118 (12.7)	1	1
	Male	671	260 (38.8)	2.9 (1.9–4.4) ^**^	2.9 (1.9–4.2) ^**^
Marital status	Married	1068	271 (25.4)	1	1
	Separated/divorced	78	23 (29.5)	1.2 (0.9–1.4)	1.2 (1.0–1.5) ^*^
	Widowed	130	20 (15.4)	0.6 (0.4- 0.9) ^*^	0.8 (0.6–1.2)
	Cohabiting	54	9 (16.7)	0.7 (0.4–1.2)	0.9 (0.5–1.5)
	Single	274	55 (20.1)	0.8 (0.6–1.0)	0.9 (0.8–1.2)
Employment	Unemployed	488	94 (19.3)	1	1
	Self-employed	933	220 (23.6)	1.2 (0.7–1.9)	0.9 (0.8–1.2)
	Employed	183	64 (35.0)	1.7 (1.1–2.8) ^*^	1.3 (0.9–1.6)

*AUD* Alcohol use disorder (AUDIT ≥ 8), *PR* prevalence ratio, *(%)* Row percentage

^*^p < 0.05

^**^p < 0.001

### Screening and management of AUD

Out of 378 participants with an AUDIT score ≥ 8, screening and management of AUD were recorded for only one participant.

### *Help-seeking behavior and barriers to seeking care among participants with AUD (AUDIT* ≥ *8)*

**Fig. 1:**
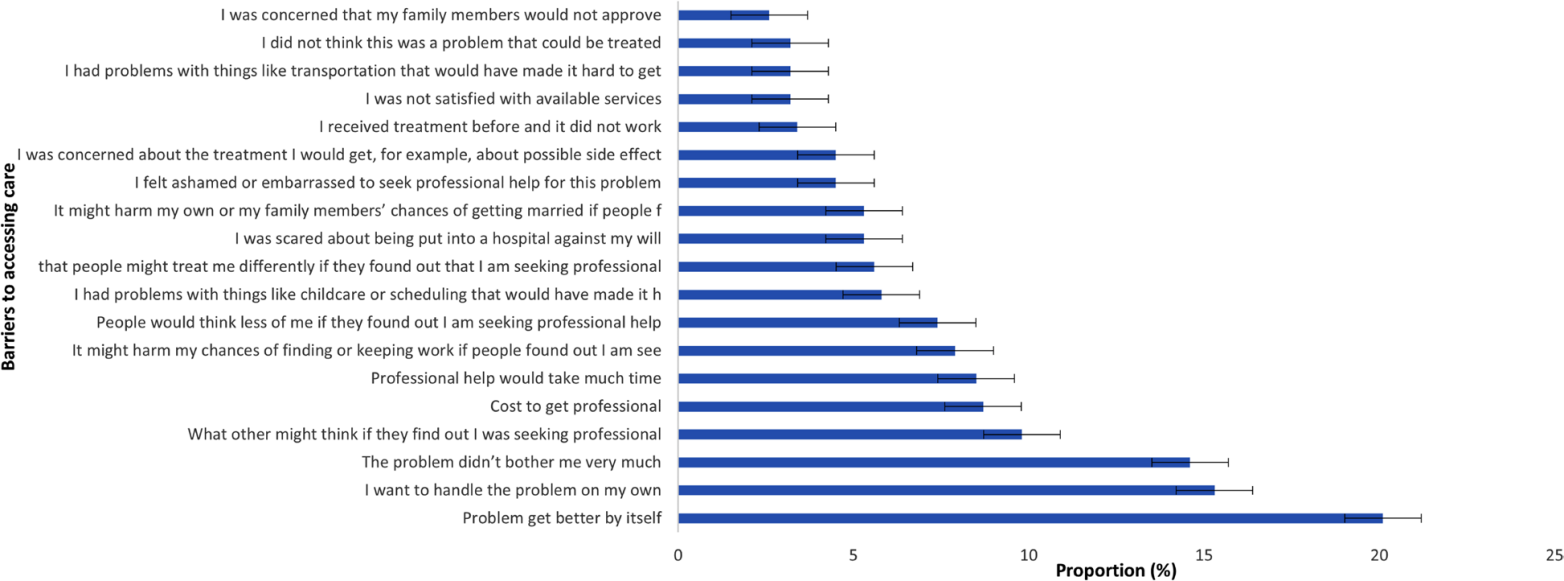
Barriers to accessing care among participants with AUDIT score ≥ 8score, figure in (%) Percentages weighted

Only 5% (20/378) had reported seeking help from informal and non-informal sources for their alcohol problem at least once in their lifetime, clan leaders/heads (*n* = 7), friends (*n* = 3), medical providers (*n* = 3), religious or spiritual leaders (*n* = 6), and a traditional healer (*n* = 1). About 148 out of 378 participants (39.1%) reported at least one barrier to seeking care for their alcohol problem. The leading reported barriers were as follows: thought that the problem would get better by itself (55.0%), wanting to handle the problem alone (42.0%), and not being bothered by the problem (40.0%). However, being not satisfied with available services (9.0%), not thinking it was a treatable problem (9.0%), and being concerned that family members would not approve (7.0%) were the least frequently reported barriers (Fig. 1).

## Discussion

This study identified a missed opportunity for detection and management of alcohol use disorder by primary health care workers, with a detection rate of 0.3%. Our study found that reported alcohol use, HED, and AUD are common among people in need of health care services in PHC facilities, although reported help-seeking behavior was low. Therefore, it is a missed opportunity to not screen alcohol use and AUD routinely among patients attending PHC.

Reported alcohol use replicates the findings from previous studies conducted in community settings in Tanzania [6, 7] and from a systematic review and meta-analysis of alcohol use in Eastern Africa [55]. However, it was higher compared to the study conducted in health care settings in Ghana [10]. The reasons could be due to the use of different screening methods for the identification of alcohol use and duration of alcohol use. Our study used the AUDIT which is self-reported and assessed the use of alcohol during the past 12 months while the previous study used a breathalyzer or saliva alcohol test strip which could detect alcohol in the body within 24 h of intake. The prevalence of HED in this study was similar to another study conducted in a clinical setting in four African countries [60]. In comparison to previous studies conducted in SSA health care settings, the prevalence of AUD in this study was akin to the study in South Africa [12] but lower than in other studies in the Eastern and Southern parts of SSA [8, 11, 61, 62]. A possible reason for the lower prevalence of AUD in the present study could be because this study was conducted in the outpatient clinics while the previous studies were conducted in HIV clinics among people living with HIV. The systematic review and meta-analysis study on the prevalence.

of AUD among people living with HIV found out that people living with HIV have a higher prevalence of AUD than the general population [56].

Alcohol use was associated with male gender and older age in line with other studies in Eastern Africa [49, 63]. Likewise, for HED [64]. In this study, the male gender was associated with an increased prevalence of AUD. The findings align with various studies in Tanzania [5, 7, 38] and in other parts of the world [17, 24, 65]. The findings are reflected in the World Health Organization’s world mental health survey cross-national epidemiology study on AUD [65] which reported that the prevalence of AUD is much higher for men than women cross-nationally. In keeping with other studies in health care facilities and community settings in SSA [17, 24, 49, 66, 67], an increase in age was positively and significantly associated with AUD.

In this study, almost no participants who screened positive for AUD were either offered an alcohol screening or any form of alcohol intervention by the health care providers. The possible reason could be that Tanzania’s guidelines have not strictly indicated a routine screening and management of AUD [68]. Moreover, this is in line with large treatment gaps for mental health disorders (including AUD) in low and middle-Income countries (LMIC) [17–19, 24, 69]. In addition to a lack of alcohol screening and brief interventions, reported help-seeking behavior for AUD services was low. Other studies in LMICs [17, 18, 24, 69], also reported low help-seeking behaviors among people attending health care facilities as well as in the community. But, the reported help-seeking behavior for the formal and informal care in this study is relatively low when compared to other studies conducted in high-income countries and that is possibly influenced by differences in socio-economic and cultural factors between high-income and low-income countries [70–72].

Several barriers to seeking care were reported by the participants in this study. The main barriers to care that were reported included, thinking that problem would get better by itself, wanting to handle the problem alone, and not being bothered by the problem. These were similar to other studies conducted in Eastern Africa [17, 24]. In comparison to another study [17], the current study did not find other reported main barriers to care such as feeling unsure about where to go and being concerned about the cost of receiving professional help. A reason for that may due to differences in the study setting. The previous study was conducted in the community while this study was conducted in healthcare facilities such that participants were already in the healthcare facilities and receiving professional help.

This study’s findings should be interpreted in light of some limitations. This study is based on self-reported information that may be predisposed to social desirability and recall bias. Therefore, participants could lead to underreporting or overreporting alcohol use, help-seeking behavior, and barriers to seeking care for AUD.

### Implications of the study

The findings call for an urgent need to integrate interventions for alcohol use and AUD services. The call is in keeping with the World Health Organization (WHO) Mental Health Gap Action Program (MhGap) for mental neurological and substance abuse (MNS) [73] The WHO MhGap guideline has emphasized integrating evidence-based interventions for AUD (including screening) in the general health care services to address the AUD treatment gap. For this to be possible the integrated interventions should equip PHC workers to detect and intervene on AUD. In addition to that, these interventions have to include strategies that will improve help-seeking behavior and address barriers to care for AUD services.

## Conclusions

While reported alcohol use, HED, and AUD are common among patients presenting to primary healthcare facilities in northern Tanzania, help-seeking behavior and detection are very low. Not screening for AUD in primary health care is a missed opportunity for early detection and management. There is an urgent need to develop interventions to increase the detection of AUD by health care providers, while also addressing help-seeking behavior and barriers to seeking care.

## Data Availability

All data used to write this paper is summarized in tables, graphs, or within text in the paper.
